# HMTM-Mediated Enhancement of Brain Bioenergetics in a Mouse Tauopathy Model Is Blocked by Chronic Administration of Rivastigmine

**DOI:** 10.3390/biomedicines10040867

**Published:** 2022-04-07

**Authors:** Renato X. Santos, Valeria Melis, Elizabeth A. Goatman, Michael Leith, Thomas C. Baddeley, John M. D. Storey, Gernot Riedel, Claude M. Wischik, Charles R. Harrington

**Affiliations:** 1 Institute of Medical Sciences, School of Medicine, Medical Sciences and Nutrition, University of Aberdeen, Aberdeen AB25 2ZD, UK; renato.santos@abdn.ac.uk (R.X.S.); v.melis@abdn.ac.uk (V.M.); e.a.goatman@abdn.ac.uk (E.A.G.); g.riedel@abdn.ac.uk (G.R.); c.harrington@abdn.ac.uk (C.R.H.); 2 Department of Chemistry, School of Natural and Computing Sciences, University of Aberdeen, Aberdeen AB24 3UE, UK; mgal.glp@abdn.ac.uk (M.L.); t.c.baddeley@abdn.ac.uk (T.C.B.); j.storey@abdn.ac.uk (J.M.D.S.); 3 TauRx Therapeutics Ltd., 395 King Street, Aberdeen AB24 5RP, UK

**Keywords:** Alzheimer’s disease, bioenergetics, hydromethylthionine, lactate, HMTM, mitochondria, rivastigmine, tauopathy, tau aggregation inhibitor

## Abstract

The tau protein aggregation inhibitor hydromethylthionine mesylate (HMTM) was shown recently to have concentration-dependent pharmacological activity in delaying cognitive decline and brain atrophy in phase 3 Alzheimer’s disease (AD) clinical trials; the activity was reduced in patients receiving symptomatic therapies. The methylthionine (MT) moiety has been reported to increase the clearance of pathological tau and to enhance mitochondrial activity, which is impaired in AD patients. In line 1 (L1) mice (a model of AD), HMTM (5/15 mg/kg) was administered either as a monotherapy or as an add-on to a chronic administration with the cholinesterase inhibitor rivastigmine (0.1/0.5 mg/kg) to explore mitochondrial function and energy substrate utilization as potential targets of drug interference. Compared with wild-type NMRI mice, the L1 mice accumulated greater levels of l-lactate and of the LDH-A subunit responsible for the conversion of pyruvate into l-lactate. In contrast, the levels of LDH-B and mitochondrial ETC subunits and the activity of complexes I and IV was not altered in the L1 mice. The activity of complex I and complex IV tended to increase with the HMTM dosing, in turn decreasing l-lactate accumulation in the brains of the L1 mice, despite increasing the levels of LDH-A. The chronic pre-dosing of the L1 mice with rivastigmine partially prevented the enhancement of the activity of complexes I and IV by HMTM and the increase in the levels of LDH-A while further reducing the levels of l-lactate. Thus, HMTM in combination with rivastigmine leads to a depletion in the energy substrate l-lactate, despite bioenergetic production not being favoured. In this study, the changes in l-lactate appear to be regulated by LDH-A, since neither of the experimental conditions affected the levels of LDH-B. The data show that HMTM monotherapy facilitates the use of substrates for energy production, particularly l-lactate, which is provided by astrocytes, additionally demonstrating that a chronic pre-treatment with rivastigmine prevented most of the HMTM-associated effects.

## 1. Introduction

The first of the current drugs approved for the treatment of Alzheimer’s disease (AD) was donepezil in 1996, followed in subsequent years by rivastigmine (2000) and galantamine (2001), all having the same mechanism of action by inhibiting acetylcholinesterase [1]. In particular, the pharmacophore of rivastigmine is its carbamate structure, which has a unique dual pseudo-irreversible inhibitor action both on acetylcholinesterase and butyrylcholinesterase, affording a more robust, transient increase in the levels of acetylcholine. After binding to acetylcholinesterase, rivastigmine is cleaved, releasing an inert product excreted in the urine, with the carbamate remaining bound to the enzyme and undergoing a much slower process of hydrolysis [2]. Memantine, approved by FDA in 2003, is being used to treat moderate to severe symptoms of AD; it has a different mechanism of action inhibiting glutamatergic neurons via extra-synaptic NMDA receptors [3]. For more than two decades, all the approved AD treatments have had only a relatively modest symptomatic efficacy over 6–9 months [4]. In 2021, the FDA approved aducanumab, an antibody targeting amyloid β (Aβ), that has been associated with some controversy arising from the absence of substantial evidence of efficacy from two phase 3 clinical trials and the negative opinion from the FDA’s own Peripheral and Central Nervous System Advisory Committee [5]. Due to contradictory results from two phase 3 clinical trials, the therapeutic benefit of aducanumab remains largely unclear and likely outweighed by side-effects, including vasogenic edema and cortical microhaemorrhages known as amyloid-related imaging abnormalities (ARIA) occurring in about 40% of participants. In addition to a lack of a clear clinical benefit, a poor post-approval marketing strategy renders aducanumab unacceptable to most clinicians, and its prescription has not been supported by health insurance agencies [6]. AD remains the most common type of dementia worldwide, having a heavy impact on quality of life for both patients and their caregivers; leaving clinicians with very limited therapeutic options, and with a poor outcome.

The association of mutations in genes related to the Aβ metabolism with familial forms of AD, which was reported in the early 1990s [7,8,9,10,11], has led to many clinical trials being undertaken with the aim of reducing Aβ deposition in the brains of patients and providing an effective disease-modifying treatment. Up until 2021, the cumulative failure of AD trials ascended to 95%, with agents targeting Aβ largely leading in number over any other individual targets, such as tau deposition or inflammatory pathways [12,13]. The failure of such trials has been disappointing for patients and clinicians, and the approach may not be able to tackle most AD cases of sporadic origin. Alternative approaches that target other neuropathological changes are yet to be fully tested.

Neurofibrillary tangles (NFTs) and their constituent paired helical filaments (PHFs) are abnormal accumulations of aberrantly folded microtubule-associated protein tau [14]. The structural cores of PHFs are composed mainly of a proteolytically stable 95-amino acid fragment of the tau protein that has the potential for self-propagation [15]. The “prion-like” processing of this short PHF-core fragment has been demonstrated in vitro [16], and the spread in the human brain during the development of AD [17] can be reproduced in a tau-transgenic mouse model expressing the core tau fragment (Line 1) [18]. Braak staging correlates closely with the decline of the cognitive function [19,20,21]. Recently, the low-density lipoprotein receptor-related protein 1 (LRP1) was found to be a crucial regulator of the propagation and aggregation of tau in the brain through an interaction with lysine residues in the microtubule-binding repeat region of tau [22]. The “prion-like” spread of PHF-tau seeds in the brain also involves the endosomal–lysosomal pathway for the release of the aggregation-prone self-propagating fragments of tau to the cytosol [23]. Importantly, in addition to the transmission between neurons, tau can be transmitted to microglia, implying that in addition to the disruption of neurotransmissions, tau aggregation also affects key brain cells involved in neuro-inflammation and the phagocytic elimination of extracellular debris [24,25]. Thus, there are several stages in tau pathology that can be targeted therapeutically to prevent the accumulation of aberrantly misfolded tau proteins [26,27].

Hydromethylthionine mesylate (HMTM) is a stable reduced crystalline form of methylthioninium (MT) that acts as a tau aggregation inhibitor (TAI) [28]. HMTM shows better pharmacodynamic and pharmacokinetic properties than the oxidised form, methylthioninium chloride (MTC, also known as “methylene blue”) [29]. The MT moiety is known to aid the mitochondrial electron transport chain (ETC) by passing electrons from ETC-reducing equivalent NADH to complexes III and IV [30,31,32] to activate cellular clearance mechanisms, such as macroautophagy [33] and ubiquitin/proteasome [34], as well as pathways related to the scavenging of reactive oxygen species (ROS) [35]. In phase 3 clinical trials testing hydromethylthionine in AD, the disease progression was slowed in patients taking HMTM as a monotherapy but not in patients already taking standard symptomatic drugs approved for the treatment of AD (acetylcholinesterase inhibitors and/or glutamatergic inhibitors) [36,37]. A population pharmacokinetic (PK) study of the participants in the clinical trials showed a steep-exposure response on clinical and brain atrophy endpoints at the 8 mg/day dose in the patients, whether they received HMTM as a monotherapy or as an add-on to symptomatic drugs, but with the treatment effects reduced by half as an add-on [38]. In a tauopathy mouse model of cognitive impairment, we have reported that the combination of symptomatic drugs with HMTM impairs treatment effects on synaptic functions, neurotransmitter releases and mitochondrial activity when compared to an administration of HMTM alone [39]. Therefore, there is a need to better understand the potential mechanisms of the interference of cholinesterase inhibitors with other drugs, including HMTM.

The brain is a highly metabolic organ, accounting for a 25% expenditure of the total circulating levels of glucose. Glucose is the main source of energy in the brain, providing metabolic intermediates for the tricarboxylic acid (TCA) cycle that fuels mitochondrial oxidative phosphorylation [40]. Glucose hypometabolism in the brain, measured using 2-deoxy-2-[^18^F]fluoro-d-glucose (^18^F-FDG) PET, has been extensively documented in the brains of AD patients [41,42,43,44,45]. The decrease in the ^18^F-FDG uptake on PET in the temporoparietal cortex is included as a sensitive determinant among the diagnostic biomarkers in recently established criteria and guidelines for AD [46]. The dysfunction of mitochondria, required for neuronal activity and neurotransmissions, is also implicated in AD [47]. In the 3xTg-AD animal model of AD [harbouring PS1(M146V), APP(Swe), and tau(P301L) transgenes], the levels of several subunits of the mitochondrial complexes I and IV were reported to be decreased [48]. Consistent with this finding, a mitochondrial bioenergetic deficit was shown to precede an AD-like pathology onset in a study conducted with female 3xTg-AD [49]. The impairment of mitochondrial bioenergetic production is known to be related to the transport of metabolites, such as pyruvate, to the mitochondrial matrix, along with their ineffective utilisation [50]. A decrease in complex I in the medial-to-lateral temporal cortex was observed recently in AD using PET [51]. A monotherapy with HMTM (8 mg/day) slowed the rate of impairment in the glucose metabolism in AD patients [37].

Given the association between a disturbed bioenergetic metabolism and the onset and progression of AD and the fact that the MT moiety enhances mitochondrial function, we aimed to determine whether the negative interaction between cholinesterase inhibitors and HMTM might be caused by changes in the oxidative phosphorylation/glycolytic profile of the brain. For this, we used a mouse model with the pathological and behavioural characteristics of AD [18] and examined the model for interactions between HMTM and rivastigmine.

## 2. Materials and Methods

### 2.1. Chemicals

All reagents used, unless otherwise stated, were obtained from Sigma-Aldrich, Dorset, UK, and were of the highest grade of purity commercially available. Rivastigmine tartrate (batch N. 1) was acquired from Tocris Bioscience, Bristol, UK. Hydromethylthionine mesylate (*N*,*N*,*N*′,*N*′-tetramethyl-10*H*-phenothiazine-3,7-diaminium bis(methanesulfonate); HMTM) (batch N. 800234810; 99.4% purity; was supplied by TauRx Therapeutics Ltd., Aberdeen, UK.

### 2.2. Transgenic Mice

Line 1 (L1) transgenic and NMRI mice were generated and bred commercially in positive pressure isolators at Charles River, UK, as described previously [18]. L1 mice express tau 296–390 of the longest human tau isoform (2N4R; htau40). The coding sequence for htau40 was fused with an N-terminal signal sequence to target the mRNA to membrane-bound ribosomes and support insertion of the signal sequence into the ER membrane [18]. Neuronal expression was ensured through insertion of the construct into the murine Thy-1 expression cassette (pTSC21k) [18].

Female homozygous L1 and NMRI mice were distributed to the Medical Research Facility, University of Aberdeen, where they were allowed to acclimatise for one month before any procedure was conducted. Animals were housed in wire-lid cages, up to 4 mice per cage, on corn cob bedding in a constant temperature and humidity environment (20–22 °C and 60–65% humidity), with 17–20 air changes per hour and 12 h light/dark cycles (lights on at 7 am). All animals were given ad libitum access to water and food pellets. Experiments were performed in accordance with the European Community Council Directive (63/2010/EU), and a project licence (number: 7008396) reviewed by the University of Aberdeen Animal Welfare and Ethical Review Body (AWERB) and approved by the Home Office on 30 January 2015. All the approved procedures were in accordance with the University Code of Practice for Research Involving the Use of Animals, compliant with the ARRIVE guidelines, and under the UK Animals (Scientific Procedures) Act (1986).

### 2.3. Experimental Design

Rivastigmine tartrate was dissolved in deionised water and administered at doses of 0.1 and 0.5 mg/kg. Stock solutions were prepared prior to the start of the experiment, and daily aliquots were stored in tightly sealed vials at −20 °C and used within 1 month. For each day of dosing, a new aliquot was brought to room temperature and mixed thoroughly before use. HMTM was dissolved daily, prior to its administration, in deionised water previously sparged with nitrogen gas for 1 h. HMTM was administered at doses of 5 and 15 mg/kg.

**Figure 1:**
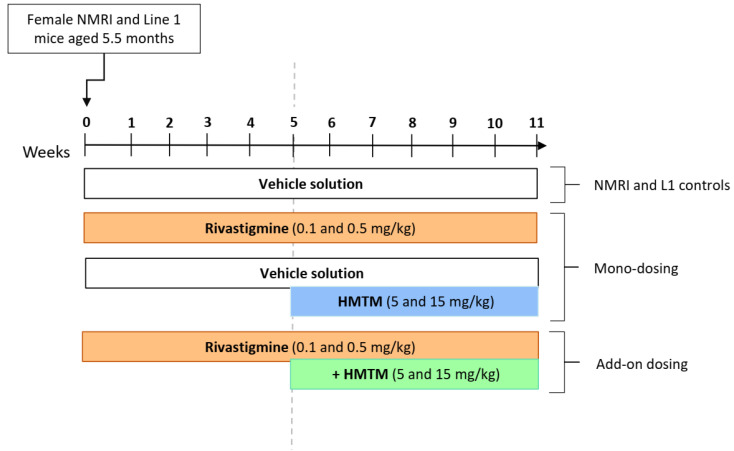
Schematic representation of the experimental design. All mice were female and aged 5.5 months at the start of the study, and the total duration of treatment was 11 weeks. NMRI mice were administered daily with vehicle or rivastigmine (0.5 mg/kg) and following that, after 5 weeks, HMTM (15 mg/kg) was added as monotherapy or add-on therapy. L1 mice were dosed with either vehicle or one of two different doses of rivastigmine (0.1 and 0.5 mg/kg) or HMTM (5 and 15 mg/kg) as monotherapy or add-on therapy.

L1 mice aged 5.5 months were weighed three times per week and gavaged daily with two doses of rivastigmine (0.1 and 0.5 mg/kg) or vehicle solution for 11 weeks. From weeks 5–11, HMTM (5 and 15 mg/kg) was also administered by oral gavage to two subgroups of mice previously gavaged with either vehicle solution (monotherapy) or rivastigmine (add-on dosing; Figure 1). Age- and gender-matched wild-type mice were also gavaged with vehicle solution, 15 mg/kg of HMTM, 0.5 mg/kg of rivastigmine, and a combination of 0.5 mg/kg of rivastigmine and 15 mg/kg of HMTM administered according to the same regime applied to L1 mice (Figure 1). During weeks 10 and 11, animals went through a behavioural training protocol (Melis et al., unpublished data). At the end of week 11, all animals were euthanised by using intraperitoneal injection of a sub-lethal dose of sodium pentobarbital, followed by blood collection by using cardiac puncture through a Plastipak syringe pre-rinsed with heparinised saline (sodium salt heparin from porcine intestinal mucosa; 10 U/mL), followed by intra-cardiac perfusion with heparinised saline solution (2–3 min). Immediately after collection, blood was transferred into plastic vials containing lithium heparin anticoagulant (Sarstedt Ltd., Leicester, UK; additive: lithium heparin; capacity: 500 µL). Blood samples were kept on ice for no more than 30 min followed by centrifugation to obtain plasma at 2000× *g* for 5 min at 6 °C (3-16KL centrifuge, Sigma, Dorset, UK). Plasma was transferred to Eppendorf tubes and kept at −20 °C until further processing. The brains were harvested and dissected sagittally, and both hemispheres were frozen separately in liquid nitrogen and then stored at −80 °C until further processing.

The study was planned and outlined prior to its implementation and registered under study reference R0139 with TauRx Therapeutics Ltd. The total number of animals in each experimental group (Table 1) varied due to the study being carried out as several consecutive replications (8 replications in total) to accommodate logistical constraints, such as the time required for the behavioural tests and tissue collection, raised by the large number of mice used in the study, which overall resulted in higher numbers of mice being used as controls. The experimenter was not blinded to the genotype, or the treatment administered to the mice, whereas randomisation was assured when distributing animals to each cage and assigning the rodents to each treatment group at the start of each study replication.

### 2.4. Extraction and Quantification of Methylthionine (MT) in the Plasma and Brains of Treated Mice

MT was extracted from samples by means of a modified dichloroethane (DCE) liquid–liquid extraction method [52]. Plasma samples (0.05 mL) were mixed with methanol, sodium hexanesulfonate (0.5%), and DCE on a rotator for 15 min, then samples were centrifuged at 18,000× *g* for 3 min at 8 °C (Hettich 220R). The right brain hemispheres were homogenised in 3× (*v*/*w*) of deionised water, followed by the addition of methanol, sodium hexanesulfonate (0.5%), and DCE. After mixing the samples for 15 min, the samples were centrifuged at 3000× *g* for 5 min at 8 °C (Fisher Accuspin 1, Fisher Scientific, Loughborough, UK).

Following centrifugation, the DCE extracts from both plasma and brain samples were collected and evaporated under vacuum at 20 °C. Samples were reconstituted with 20 mM of ammonium bicarbonate: acetonitrile (60:40, *v*/*v*).

The reconstituted samples were analysed for MT by using UPLC with LC-MS/MS detection. MT is a redox molecule, and depending on environmental conditions, such as pH, oxygen, and reducing agents, it exists in equilibrium between a reduced (leucomethylthioninium; LMT) and oxidised form (MT^+^). The MT moiety is ionised by the mass spectroscopy procedure; hence, LMT and MT^+^ forms in the samples were not distinguished. The lower limits of detection for MT in plasma and brains were 0.05 and 1 ng/mL, respectively.

### 2.5. Tissue Processing for Cell Biology and Metabolite Analysis

The left hemisphere of each mouse was subdivided and subjected to differential processing according to the following procedures. An adult mouse brain matrix with sagittal 1-mm thick clefts and obtained from Agntho’s AB (Lidingö, Sweden) was used to obtain replicable sections of brain tissue from each individual sample, corresponding to approximately one quarter of the hemisphere. Each of the tissue sections was homogenised, according to the subsequent detailed description, for immunoblotting, enzyme activities, and metabolite quantification.

### 2.6. Total Protein Extraction for Immunoblotting

The tissue was homogenised in ice-cold RIPA buffer 1×, diluted from 10× stock (Cell Signaling Technology, Danvers, MA, USA), and supplemented with cOmplete^TM^ protease inhibitor cocktail tablets (Roche, Mannheim, Germany), phosphatase inhibitors (cocktail 2 and cocktail 3), and phenylmethylsulfonyl fluoride (PMSF; 1 mM). Homogenisation was performed with a hand-held homogeniser and disposable polypropylene pestles. After homogenisation, the samples were maintained at 4 °C for 90–120 min with rotation. Samples were then further disrupted by snap-freezing in liquid nitrogen three times. Lastly, samples were centrifuged at 14,000× *g* (PK131R centrifuge, ALC Int., Milan, Italy) for 10 min at 4 °C, and the supernatant fractions were collected and stored at −80 °C for use in immunoblot analysis.

### 2.7. Protein Extraction for Determination of Enzyme Activities (Citrate Synthase; NADH Dehydrogenase; Cytochrome c Oxidase)

The tissue was homogenised in ice-cold Tris-buffered solution (10 mM; pH 7.5) containing n-dodecyl-β-D-maltoside (125 mM). Homogenisation was performed with a hand-held homogeniser and disposable polypropylene pestles. After homogenisation, the samples were maintained at 4 °C for 90–120 min with rotation. Samples were then further disrupted with 7–10 strokes using a 25-gauge syringe needle. Lastly, samples were centrifuged at 14,000× *g* (PK131R centrifuge, ALC Int.) for 10 min at 4 °C and supernatants were collected and stored at −80 °C for determination of enzymatic activities. 

### 2.8. Tissue Processing for l-Lactate Quantification

The tissue was homogenised in ice-cold lysis buffer 1×, diluted from a 10× stock (Cell Signaling Technology, Danvers, MA, USA) and supplemented with cOmplete^TM^ EDTA-free protease inhibitor cocktail tablets (Roche), phosphatase inhibitors (cocktail 2 and cocktail 3), and phenylmethylsulfonyl fluoride (PMSF; 1 mM). Homogenisation was performed with a hand-held homogeniser and disposable polypropylene pestles. After homogenisation, the samples were maintained at 4 °C for 90–120 min with rotation. Samples were then further disrupted by snap-freezing in liquid nitrogen three times. Lastly, samples were centrifuged at 14,000× *g* (PK131R centrifuge, ALC Int.) for 10 min at 4 °C, and the supernatant fractions were collected and stored at −80 °C for quantification of l-lactate.

### 2.9. Immunoblot Analysis

The protein content in the sample extracts were determined using the bicinchoninic acid (BCA) protein assay using the BCA kit (Pierce Thermo Fisher Scientific, MA, USA). The samples (15–30 μg protein per lane, depending on the protein to be labelled) were resolved by using electrophoresis in 8–16% TGX pre-cast midi gels (Bio-Rad, Hercules, CA, USA) and transferred to polyvinylidene difluoride (PVDF) midi-sized membranes. Non-specific binding was blocked by slowly rotating the membranes in 5% BSA and 0.1% Tween^®^ 20 in Tris-buffered saline (TBS) for 90–120 min at room temperature. The blots were then incubated with specific primary antibodies overnight at 4 °C with slow rotation. The primary antibodies used were the total rodent antibody OXPHOS cocktail (Abcam, Cambridge, UK), for detection of subunits of the complexes of the mitochondrial respiratory chain, and mouse monoclonal IgGs anti-LDH-A and anti LDH-B (Proteintech, Manchester, UK) for the detection of the lactate dehydrogenase (LDH) subunits. Blots were washed three times (3 × 15 min) with TBS containing 0.1% Tween^®^ 20 (TBS-T) and then were incubated with secondary antibody (HRP-conjugated anti-mouse IgG, Bio-Rad) for 90–120 min at room temperature with slow rotation. After three washes with TBS-T, specific bands of immunoreactive proteins were visualised using enhanced chemiluminescence (ECL SuperSignal™ West Dura Extended Duration Substrate, Pierce Thermo Fisher Scientific, Waltham, MA, USA) with varying exposure times in a BioSpectrum 810 Imaging System (UVP BioImaging Systems, Upland, CA, USA). The density of protein bands was calculated using the UVP Vision Works LS 8.2 (Life Science Software from UVP) and normalised for the density of total proteins stained with MemCode^TM^ reversible protein stain kit (Pierce Thermo Fisher Scientific, Waltham, MA, USA); this was performed following standard procedure for each membrane.

### 2.10. Citrate Synthase Activity

As an indicator of mitochondrial content in each sample, citrate synthase activity was measured using a colorimetric assay kit (Biovision, Upland, CA, USA), according to the manufacturer’s instructions. Briefly, citrate synthase reacts with a mixture of substrates, producing an intermediate that then reacts with the development reagent, generating a coloured product. Total protein (50 µg) was used in the assay with the final volume in each well up to 100 µL with assay buffer. The rate of change in absorbance (Spectramax 190, Molecular Devices, San Jose, CA, USA) was measured for 40 min at 25 °C, and the activity of the enzyme was calculated based on the molar extinction coefficient of 5,5′-dithiobis-(2-nitrobenzoic acid) (ε = 6.8 mM^−1^) at 412 nm.

### 2.11. NADH Dehydrogenase Activity

The activity of NADH dehydrogenase (mitochondrial complex I) was determined with a complex I enzyme-activity microplate colorimetric-assay kit (Abcam, Cambridge, UK) according to the manufacturer’s instructions. Briefly, whole tissue extract (50 µg of protein) was loaded onto each well of the 96-well plate, bringing up the volume to 200 µL with 1× incubation buffer. The microplate was incubated for 3 h at room temperature to allow complex I to be immunocaptured by antibodies pre-coated on plate wells. Complex I activity was measured following the oxidation of NADH to NAD^+^ with simultaneous reduction of the supplied dye and absorbance at 450 nm measured for 30 min using a Spectramax 190 plate reader (Molecular Devices, San Jose, CA, USA). Complex I activity is proportional to the rate of change in absorbance and calculated as a measure of the reduction of the supplied dye using its extinction coefficient (ε = 25.9 mM^−1^) and normalised per mg of total protein. Overall activity of complex I was, ultimately, normalised to activity units of citrate synthase as a measure of the mitochondrial content of each sample.

### 2.12. Cytochrome c Oxidase Activity

The activity of mitochondrial cytochrome c oxidase (mitochondrial complex IV) was determined with a complex IV enzyme-activity microplate colorimetric-assay kit (Abcam, Cambridge, UK) according to manufacturer’s instructions. Briefly, whole tissue extract (50 µg of protein) was loaded onto each well of the 96-well plate, bringing up the volume to 200 µL with 1× incubation buffer. The microplate was incubated for 3 h at room temperature to allow complex IV to be immunocaptured by the antibodies pre-coating the plate wells. Complex IV activity was measured following the oxidation of reduced cytochrome c for 40 min at 30 °C, reflected in a decrease in absorbance at 550 nm (Spectramax 190, Molecular Devices, San Jose, CA, USA). Complex IV activity is a function of the change in the rate of absorbance, calculated as a measure of the decrease in reduced cytochrome c using its extinction coefficient (ε = 7.04 mM^−1^) and normalised per mg of total protein. Overall activity of complex IV was, ultimately, normalised to activity units of citrate synthase as a measure of the mitochondrial content of each sample.

### 2.13. l-Lactate Quantification

For determination of ʟ-lactate levels in the brains of mice, proteins in the tissue extractions were inactivated by using an addition of 1/8 of the used sample volume of HCl (0.6 M) and, after 1 min, neutralised with Tris solution (1 M; same volume as HCl). Inactivation of proteins is intended to eliminate interference from the activity of endogenous dehydrogenases in the detection of l-lactate. After neutralisation, the pH of each sample was checked using pH strips. This method for inactivation does not form precipitates; therefore, there is no need to centrifuge the samples. The quantification of the amount of ʟ-lactate in the samples was performed according to manufacturer’s instructions using a bioluminescent Lactate-Glo^TM^ assay (Promega, Madison, WI, USA). Briefly, 25 µL of previously inactivated samples were loaded into white 96 half-area microplate. Reaction mixture (25 µL) containing LDH, NAD, ATP, and pro-luciferin reductase substrate was added, and plate was incubated for 60 min before reading luminescence using a CLARIOstar Plus multimode microplate reader (BMG Labtech, Ortenberg, Germany). The amount of l-lactate was determined using a standard curve and normalised per mg of protein.

### 2.14. Statistical Analysis

The statistical analysis was conducted using R [53]. NMRI and L1 vehicle-gavaged mice were compared for the outcome measures ʟ-lactate, LDH-A, LDH-B, NDUFB8-CI, SDHB-CII, UQCRC2-CIII, MTCOI-CIV, ATP5A-CV, complex I activity, and complex IV activity. Summary statistics were calculated, and differences between genotypes were investigated with a Wilcoxon rank sum test (normality assumptions for parametric models did not hold under the Shapiro–Wilks test). For the L1 mice cohort, differences in MT concentrations in brain tissues across treatment groups were explored using a linear model. Examination of the model residuals, including the Shapiro–Wilks test, confirmed the appropriateness of the parametric model. Correction for multiple comparisons was made using Tukey’s test [54]. For L1 mice, it was found that the data for the remainder of the outcome measures (ʟ-lactate, LDH-A, LDH-B, complex I activity, and complex IV activity) did not satisfy criteria for parametric modelling for effects of treatment group. Therefore, non-parametric models were fitted; the first was Kruskal–Wallis to determine any treatment effects. In the case of a significant treatment effect, this was followed by a post hoc Dunn’s test to determine differences between treatment groups and correct for multiple comparisons [55]. For all tests, differences associated with a *p* value < 0.05 (following multiple comparison corrections where necessary) were considered significant.

## 3. Results

### 3.1. L1 Tau-Transgenic Mice Show Accumulation of l-Lactate in the Brain

For the characterisation of the metabolic profile of the brains of the L1 mice compared with their genotypic-background NMRI controls, we quantified the levels of ʟ-lactate and subunits A (astrocytic) and B (neuronal) of the lactate dehydrogenase enzymes, as well as the mitochondrial complex subunits of the ETC. The tau-transgenic L1 mice showed significantly higher levels of ʟ-lactate, accompanied by higher levels of LDH subunit A but not subunit B (Table 2). From the assessment of the oxidative phosphorylation-related parameters, no differences were observed between the L1 and wild-type NMRI brains in the levels of the subunits NADH:ubiquinone oxidoreductase subunit B8 (NDUFB8, complex I); succinate dehydrogenase complex iron-sulphur subunit B (SDHB, complex II); ubiquinol-cytochrome c reductase core protein 2 (UQCRC2, complex III); mitochondrial cytochrome c oxidase subunit 1 (MTCOI, complex IV) and ATP synthase F1 subunit alpha (ATP5A, complex V; Table 2). Similarly, the enzymatic activity of the mitochondrial complexes I and IV remained unchanged in the L1 mice when compared to the NMRI brains (Table 2). Thus, the L1 mouse model displayed characteristics of the metabolic impairment of the brain, validating it as a suitable and relevant model for the study of drug interference of brain metabolism through a tau-related mechanism.

### 3.2. Activity of ETC Complex I and Complex IV Is Facilitated by HMTM

**Figure 2:**
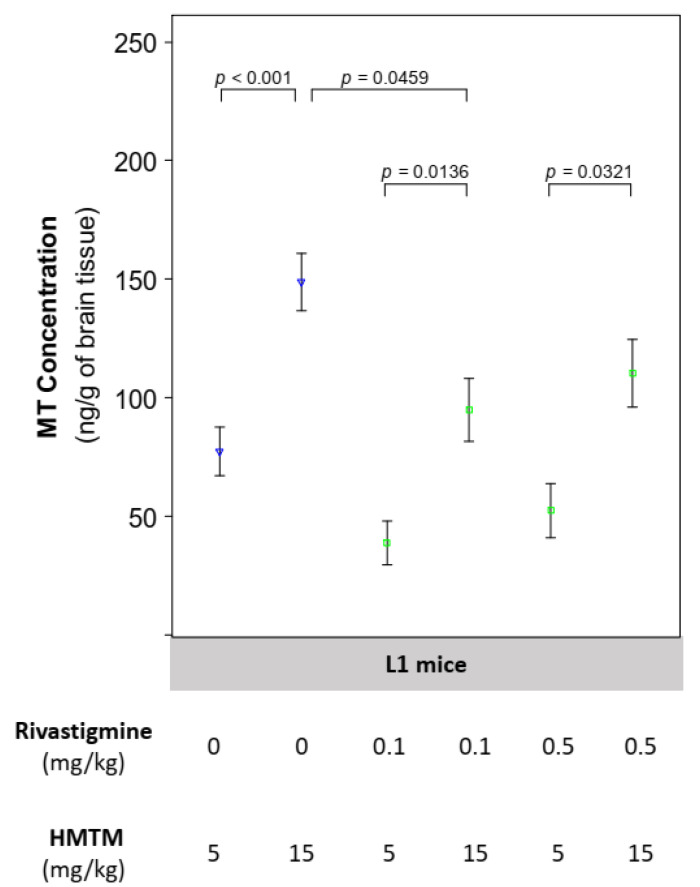
Brain concentrations of MT in L1 mice. For L1 mice administered solely HMTM at 15 mg/kg, there was a significant doubling in the brain concentration of MT when compared to mice injected with 5 mg/kg (*p* < 0.001). When HMTM was co-administered with rivastigmine, the brain concentration of MT was decreased (*p* = 0.0459, L1 rivastigmine 0.1 mg/kg + HMTM 15 mg/kg vs. L1 HMTM 15 mg/kg). Nevertheless, the proportional increase between both HMTM doses was maintained independently of the dose of rivastigmine being administered (0.1 mg/kg, *p* = 0.0136; 0.5 mg/kg, *p* = 0.0321). Increasing the dose of rivastigmine from 0.1 to 0.5 mg/kg produced no difference between L1 mice receiving the same dose of HMTM. Data are expressed as mean values ± SEM (*n* = 5–12). Differences are considered statistically significant when *p* < 0.05. Regardless of the doses, the blue symbols represent the experimental groups dosed with HMTM; and the green symbols represent the groups dosed with both HMTM and rivastigmine.

To determine whether HMTM dosing alone or combined with rivastigmine had an effect on mitochondrial function, we assayed the activity of complex I and IV following the oxidation of their corresponding specific substrates. We also measured the brain levels of MT, known to be the relevant active moiety of HMTM, with an oxidation-reduction potential of 10 mV, mid-ranging the ETC redox potentials (−0.4 V to 0.4 V), thus having the potential to chemically aid the electron transport through the ETC. In the brains of the NMRI mice, HMTM and rivastigmine did not induce any noticeable changes in the activity of either complex I or complex IV whether alone or in combination (Figure S1A,B). In the brains of the L1 mice, the 15 mg/kg dose of HMTM produced higher brain levels of MT both alone and in combination with rivastigmine compared with the 5 mg/kg dose (Figure 2). However, the co-administration with rivastigmine attenuated the HMTM dose-dependent increase in brain MT at both doses of rivastigmine (0.1 and 0.5 mg/kg) when compared to the equivalent administration doses of HMTM in a single regime; this was more evident at the HMTM dose of 15 mg/kg (Figure 2). Similarly, in the brains of the NMRI mice, rivastigmine also decreased the concentration of MT (Figure S2). To determine whether there were changes in the activity of mitochondrial complexes due to differences in the brain bioavailability of MT when combined with rivastigmine, we pooled the data for the two doses of rivastigmine for the combination experiments with HMTM.

**Figure 3:**
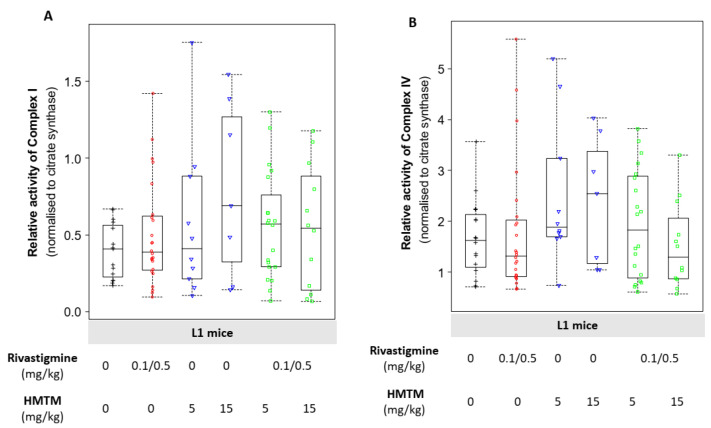
Relative levels of mitochondrial complex I (**A**) and complex IV (**B**) activity in the brains of L1 tau-transgenic mice under dosing regimens of HMTM given singly or as an add-on to rivastigmine administration. HMTM alone, not in conjunction with rivastigmine, increased the levels of mitochondrial complex I (**A**) and complex IV (**B**). Activities are normalised to the activity of citrate synthase (CS) and data are expressed as median values ± IQR (*n* = 7–24). Regardless of the doses, the black symbols represent the vehicle group; the red symbols represent the rivastigmine-dosed mice; the blue symbols represent the experimental groups dosed with HMTM; and the green symbols represent the groups dosed with both HMTM and rivastigmine.To understand whether the changes observed in the activity of both complexes reflected changes in the levels of the functional subunits of the ETC or were due to changes in functional activity, we undertook the immunolabelling of the individual components of the mitochondrial complexes.

At a dose of 15 mg/kg, HMTM increased complex I activity normalised to citrate synthase levels, although the increase did not reach statistical significance (Figure 3A). Such an increase was halted when HMTM was given as an add-on to rivastigmine to the L1 mice (Figure 3A). Likewise, HMTM produced a more consistent increase in the activity of complex IV when compared with the animals dosed with the vehicle solution than the brains of the mice receiving HMTM in combination with rivastigmine (Figure 3B). Thus, these analyses showed that HMTM given alone potentiated the mitochondrial ETC function in the brains of the L1 mice in a dose-dependent manner. When rivastigmine was dosed chronically prior to and with HMTM, it prevented the ability of HMTM to enhance the activity of the mitochondrial complexes, particularly complex IV activity.

### 3.3. Single and Combined Dosing of Rivastigmine and HMTM Do Not Change the Levels of Subunits of the Mitochondrial Electron Transport Chain

No noticeable differences were reported between the vehicle-dosed NMRI and L1 transgenic mice in the levels of the subunits NDUFB8 (complex I; Figure S3A,F), SDHB (complex II; Figure S3B,F), UQCRC2 (complex III; Figure S3C,F), MTCOI (complex IV; Figure S3D,F), and ATP synthase F1 subunit alpha (ATP5A; complex V; Figure S3E,F). Additionally, none of the drugs or treatment combinations produced any shift in the levels of any of the ETC-constituent subunits of mitochondrial complexes I to V measured in the NMRI mice (Figure S3A–F). Similarly, neither rivastigmine nor HMTM, dosed singly or together, elicited detectable changes in any of the proteins of the complexes (Figure S3A–F). Since the protein levels of the structural subunits of the ETC were not altered by genotype or drug administration, as opposed to ETC complexes I and IV functional activity, we next sought to explore how transgenes and treatments affected the metabolic l-lactate astrocyte-neuron shuttle pathway, known to be a source of a mitochondrial energy substrate.

### 3.4. HMTM-Induced Reduction in the Levels of Brain l-Lactate Is Lowered Further by the Prior Chronic Administration of Rivastigmine

**Figure 4:**
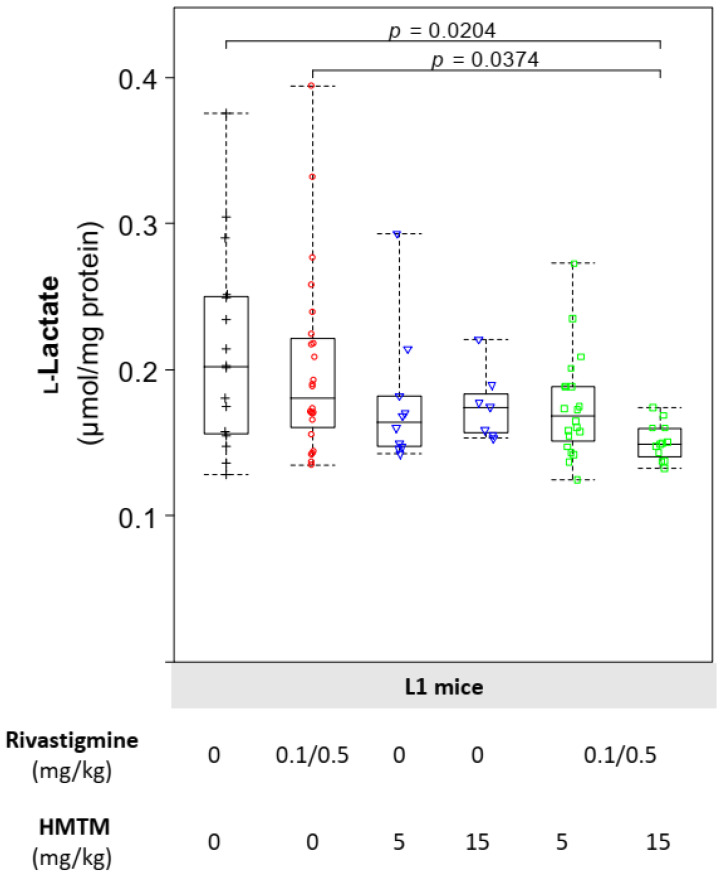
Levels of l-lactate in the brain tissue of L1 mice. For mice under the add-on regimen at the higher concentration of HMTM (15 mg/kg), the levels of l-lactate in the brain of L1 mice were significantly decreased. Data are expressed as median values ± IQR (*n* = 7–24). Differences are considered statistically significant when *p* < 0.05. Regardless of the doses, the black symbols represent the vehicle group; the red symbols represent the rivastigmine-dosed mice; the blue symbols represent the experimental groups dosed with HMTM; and the green symbols represent the groups dosed with both HMTM and rivastigmine.

l-Lactate in the brain is partially derived from astrocytes through a breakdown of their glycogen storage, and this local reserve of energy plays an important role in memory formation and consolidation [56,57]. Neither rivastigmine nor HMTM, neither singly nor together, showed any effect on the l-lactate levels in the NMRI mice (Figure S4). In the tau-transgenic L1 mice, rivastigmine did not produce any changes in the levels of l-lactate in the brains when compared to the L1 vehicle group (Figure 4). Both the low (5 mg/kg) and the high (15 mg/kg) doses of HMTM produced clear tendencies to decrease the brain levels of l-lactate when compared either to the respective L1 vehicle-injected animals (Figure 4). The L1 mice co-administered with rivastigmine and the high dose of HMTM had significant reductions in their brain levels of l-lactate when compared to the L1 mice given the vehicle or chronically dosed with rivastigmine alone (Figure 4). Therefore, rivastigmine combined with HMTM is responsible for a decrease in this important CNS reserve of energy substrate, known to be produced in the astrocytes. Such a decrease in the brain level of l-lactate may result from either a reduction in the reserve of the precursor glycogen/pyruvate, a decrease in the astrocytic LDH-A-mediated rate of conversion of pyruvate into l-lactate, and/or an increase in the neuronal LDH-B-mediated conversion of l-lactate into the acetyl-CoA precursor pyruvate [58,59,60]. To further explore how treatment was affecting the brain l-lactate metabolism in the tau-transgenic mice, while addressing what could be a dual cell-specific effect, we proceeded to evaluate the levels of LDH-A and LDH-B.

### 3.5. HMTM Monotherapy Increases the Levels of Astrocytic LDH-A Subunits

LDH is a tetrameric enzyme composed of different combinations of subunits A and B. Functionally, B subunits, primarily neuronal, convert l-lactate into pyruvate, whereas A subunits, primarily astrocytic, convert pyruvate into l-lactate [61].

**Figure 5:**
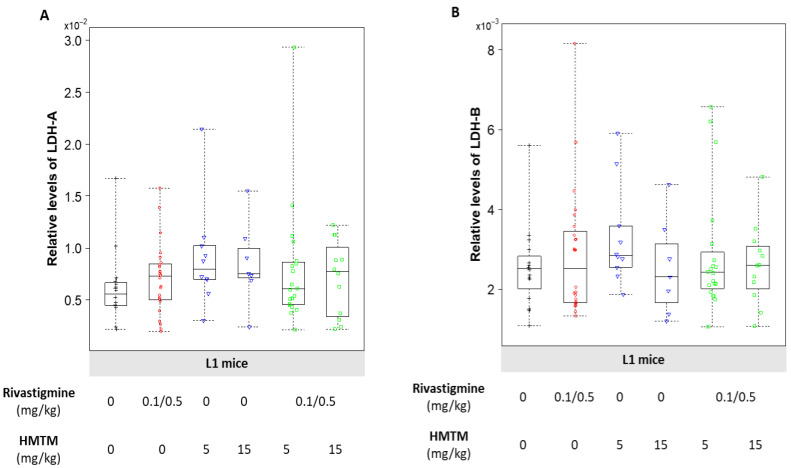
Relative levels of LDH-A (**A**) and LDH-B (**B**) in the brains of L1 mice. HMTM tended to increase the levels of LDH-A in the brains of L1 mice, with rivastigmine preventing the same profile of increase in the LDH-A subunit (**A**). HMTM dosing as either mono- or add-on regimen of administration failed to reveal any changes in the protein levels of LDH-B (**B**). Data are expressed as median values ± IQR (*n* = 7–24). Regardless of the doses, the black symbols represent the vehicle group; the red symbols represent the rivastigmine-dosed mice; the blue symbols represent the experimental groups dosed with HMTM; and the green symbols represent the groups dosed with both HMTM and rivastigmine.

In the L1 mice, HMTM showed a clear tendency to increase the levels of LDH-A, with the levels of protein being similar for both doses of HMTM given singly (Figure 5A). When both rivastigmine and HMTM were given in combination to the L1 mice, the levels of LDH-A showed a tendency to decrease to levels similar to those observed in the L1 mice given the vehicle (Figure 5A). The levels of LDH-B were largely unchanged for all the treatment conditions of the L1 mice (Figure 5B). Despite evoking a similar increase in the relative levels of both LDH-A and LDH-B in the NMRI mice when rivastigmine and HMTM were dosed alone, the drugs showed a tendency to increase the expression levels of both LDH subunits when they were given together (Figure S5A,B).

The effects of HMTM on the l-lactate levels and metabolism in the brains of the tau-transgenic mice appeared to be associated with a change in composition of the LDH subunits favouring an increase in LDH-A, that is, at least partially prevented in the context of the rivastigmine co-administration. Thus, the effect of HMTM on brain metabolism may have a dual cell-specific role favouring the astrocyte conversion of pyruvate into l-lactate by increasing LDH-A subunits, provisioning an unaffected neuronal LDH-B with pyruvate precursor and ultimately fueling an MT-facilitated electron transport through the ETC and, in turn, oxidative phosphorylation. When preceded by the chronic administration of rivastigmine, such an HMTM-facilitated metabolic conversion of substrates into bioenergy by mitochondrial oxidative phosphorylation is decreased at the level of the astrocytic production of l-lactate by LDH-A and mitochondrial function.

## 4. Discussion

From a post hoc analysis of an 18-month randomised, controlled double-blind phase 3 clinical trial enrolling a total of 891 AD patients, it was concluded that HMTM was effective as a monotherapy, suggesting that comedication with classical symptomatic therapies would affect the efficacy of HMTM [36,37]. ^18^F-FDG-PET was used in this trial to confirm that the rate of deterioration in the brain glucose metabolism was slowed in patients receiving HMTM as a monotherapy compared with those receiving HMTM as an add-on therapy [37]. To understand the basis for the changes in glucose metabolism observed in the clinic, we designed a mouse-model study to investigate the effects of HMTM on the metabolic pathways that might be impacted by anticholinesterase inhibitors. Since HMTM is a tau aggregation inhibitor (TAI), we used, as an animal model, the L1 mice, which overexpress the truncated 12-kD, 95-amino acid tau fragment that constitutes the core tau protein of the paired helical filaments (PHFs) in AD [18,62,63]. We first characterised tau transgenic L1 mice with the NMRI wild-type background and then investigated the effects of HMTM, alone and combined with rivastigmine, for two interconnected metabolic pathways that are critical for the brain energy demands that are known to be impaired in AD: l-lactate metabolism/astrocyte-neuron lactate-shuttle and mitochondrial function.

Glucose metabolism is the main source of energy in the brain, providing the reducing equivalents, via the TCA cycle, that fuel the mitochondrial ETC. This accounts for an estimated 95% contribution to ATP production, with the human brain consuming around 20% of total energy requirement of the body, even though it represents only 2% of the total body mass [64]. The processes related to synaptic transmission consume most of this energy with excitatory neurons consuming 80–85% of brain ATP and inhibitory neurons accounting for the remainder [64]. This further emphasises the physiological significance of a balanced and fully functional bioenergetic machinery to fulfil the requirements of brain function. Whereas neurons have high rates of oxidative phosphorylation as the most efficient way to meet their energy demands, astrocytes have a comparatively highly glycolytic profile, being key in the production and release of **l**-lactate as well as in the accumulation of the reserve substrate glycogen [65,66]. Thus, **l**-lactate is a key metabolite in the brain, in which astrocytes function as trophic support cells with neurons meeting their energy requirements. The L1 mice had a significantly greater accumulation of l-lactate and higher levels of LDH-A in the brain when compared with the wild-type NMRI mice (Table 2). The levels of LDH-B and measures of mitochondrial impairment did not differ between the different mouse strains (Table 2). Our previous work demonstrated the efficacy of HMTM in decreasing the accumulation of abnormal tau, particularly in the hippocampus and entorhinal cortex, and rescuing learning impairments in L1 mice [67]. We have now extended our understanding of the pharmacological action of HMTM in ways that might account for the potential interference of HMTM activity by rivastigmine (0.1 and 0.5 mg/kg). The efficacy of HMTM to reduce the immunoreactivity of the tau labelling of neurons in the vertical diagonal band of the Broca of L1 mice was also affected negatively by rivastigmine [39]. While HMTM decreased the brain accumulation of l-lactate in the L1 mice, such a decrease was exacerbated at the higher dose of HMTM in the presence of rivastigmine (Figure 4). As a mono- and add-on dose, the HMTM-induced decrease in accumulation of l-lactate in the brain is likely due to increased levels of the subunit A of the enzyme LDH (Figure 5A). Our results also indicate that HMTM facilitates mitochondrial function, as demonstrated by an increase in the activity of mitochondrial complexes I and IV, an effect partially prevented by the co-administration of HMTM with rivastigmine (Figure 3A,B).

### 4.1. The Concentration of Brain MT Increases with Dose of HMTM and Is Reduced by Its Concomitant Administration with Rivastigmine

The quantification of the active moiety of HMTM in the brain tissues demonstrated that the higher dose of HMTM (15 mg/kg) resulted in a greater bioavailability of MT in the brain when compared to the lower dose of HMTM (5 mg/kg) when administered alone or when administered in combination with rivastigmine (Figure 2). The presence of rivastigmine, however, reduced the amount of MT in the brains of the L1 mice regardless of the HMTM dose, with both concentrations of rivastigmine (0.1 and 0.5 mg/kg) resulting in similar reductions in the bioavailability of MT (Figure 2). For this reason and due to the limitation in the number of animals in the experimental groups dosed with both HMTM and rivastigmine, we clustered the biological data from the combination dosing groups, considering only high (15 mg/kg) and low (5 mg/kg) doses of HMTM regardless of the concentration of rivastigmine. Aside from increasing the statistical power, this analysis strengthened our conclusions by emphasising the effect of HMTM dosing based on the distribution of the active moiety in the absence and presence of rivastigmine. In a pharmacokinetic analysis of the plasma from 1296 patients enrolled in two phase 3 clinical trials, the comedication of HMTM with acetylcholinesterase inhibitors and/or memantine did not affect the plasma concentration of the active moiety [38]. Although we do not know the brain concentrations of MT in these AD subjects, we can note that rivastigmine also did not interfere with the plasma MT concentrations in our mice (Figure S6), thus mirroring the pharmacokinetic data from the clinic.

### 4.2. L1 Mice Are Relevant Models of Tauopathy for the Study of l-Lactate Metabolic Alterations despite Unchanged Mitochondrial Function

The L1 mice showed an increased accumulation of l-lactate in the brain (Table 2), a feature shared commonly with animal models of accelerated aging [59] and with fibroblasts obtained from late-onset AD patients [60]. In addition to its AD-like tau pathology [18] and cholinergic deficit [68], this provides a further feature that makes L1 mice a relevant pre-clinical model for AD. Accordingly, the levels of LDH-A were also increased in L1 mice (Table 2). LDH subunits A are the sole component of the predominantly astrocytic homotetrameric isoenzyme LDH5, whereas subunits B assemble the neuronal homotetramer LDH1. Functionally, LDH-B catalyses the conversion of l-lactate into pyruvate and LDH-A converts pyruvate into l-lactate [69]. Thus, astrocytes possess a glycolytic capacity, that is lacking for neurons. Hence, a neuronal overexpression of the aggregating truncated PHF-core tau domain is associated with an increase in l-lactate in L1 brains, at least in part explained by an increased capacity for the conversion of pyruvate into l-lactate. l-Lactate should be able to fuel a fully functional mitochondrial ETC to satisfy the energy requirements for synaptic transmission. The L1 mice did not show alterations in the levels of complexes I to IV, ATP synthase, or the activity of complexes I and IV (Table 2). Despite alterations in the protein levels of mitochondrial ETC subunits, as well as functional changes, not being observed in our L1 mouse model, proteomic and functional analyses carried out on other AD animal models, such as 3xTg-AD, have reported changes in the subunits of the ETC (such as the components of mitochondrial complex I and complex IV), mitochondrial respiration [48], and complex V subunit ATP5H [70]. The complexity of the animal model and the ages and genders of the mice may account for the varying observations of mitochondria. Nonetheless, the pathological connections between aberrant tau depositions and aggregations and mitochondria impairment have been well-documented. Tauopathy-related changes in mitochondrial bioenergy production; the homeostasis of second messengers (reactive oxygen species and Ca^2+^ levels) and apoptosis resulting from and exacerbated by the impaired intracellular movement of mitochondria; and the dynamic changes of fission/fusion events and ultrastructural misshaping have all been described [71,72,73,74]. Ultrastructurally, a truncated tau protein has been colocalised with abnormal mitochondria, and mitochondrial proteins copurify with proteolytically stable PHFs [15,75]. In humans, Terada and colleagues [51] used PET scans to reveal a reduced labelling of complex I in the medial temporal cortices of AD patients.

Overall, despite no evident mitochondrial disturbance, the L1 mice displayed relevant metabolic alterations in the context of aging and AD, and these are likely to lead to a bioenergetic imbalance.

### 4.3. HMTM Monotherapy Enhances Energy Metabolism by Increasing Substrate Supply to More Active Mitochondria

Out of approximately 90 subunits of the ETC complexes, 85% are synthesised in the cytosol, with the remainder in the mitochondrial matrix [76]. Only complex II is completely encoded by nuclear genes, and it is the only one not pumping protons to the intermembrane space [77]. We investigated the levels of the structural subunits belonging to all the ETC complexes and did not observe any HMTM-induced differences in the levels of critical subunits for any of the complexes in the respiratory chain (Figure S3A–F). MTC, the oxidised crystalline form of MT, enhances mitochondrial activity at low concentrations (10–100 nM) by acting as a supplementary electron carrier in the ETC [30,31,32]. Here, we report that HMTM alone increased the activity of mitochondrial complexes I and IV in L1 mice, and the effect was more prominent at a higher dose of HMTM (15 mg/kg; Figure 3).

The tendency of HMTM to increase the levels of LDH-A indicates the potential for the increased conversion of pyruvate to l-lactate in astrocytes, which contrasts with the overall observation that HMTM treatments decrease the levels of l-lactate in the brain. The net decrease in l-lactate, despite increased LDH-A levels, and in addition to the enhancement of the activity of the mitochondrial ETC complexes I and IV are together likely to lead to an overall improvement of brain bioenergetics. Alternatively, it is plausible to hypothesise that the seemingly paradoxical decrease in the levels of l-lactate, despite the increase in the levels of LDH-A, derives from a change in the levels of protein that is not reflected by enzyme functionality, similar to what happened with the unchanged levels of mitochondrial ETC subunits despite an increase in the function of mitochondrial complexes I and IV.

### 4.4. HMTM Administered as an Add-On Drug to Mice Chronically Dosed with Rivastigmine Fails to Restore the Brain Energetic Profile in the L1 Transgenics

In the presence of rivastigmine, HMTM failed to enhance the activity of mitochondrial complexes I and IV. The add-on dosing did not increase the levels of LDH-A and decreased the concentration of l-lactate in the brains of the L1 tau-transgenic mice. In addition to the failure of the add-on therapy to improve mitochondrial activity, this situation culminated in the failure of both the local supply of substrates and of the machinery that generates cell energy. Overall, we provide evidence to support the suggestion that a system energetically exhausted by the prolonged administration of cholinesterase inhibitors as a consequence of the futile and energetically inefficient use of reserves of metabolic substrates or simply due to the rivastigmine-related reduction of the bioavailability of the active MT-moiety fails to respond to hydromethylthionine, which, on its own, can enhance brain bioenergetics in addition to its action in reducing the burden of aggregated tau.

**Figure 6:**
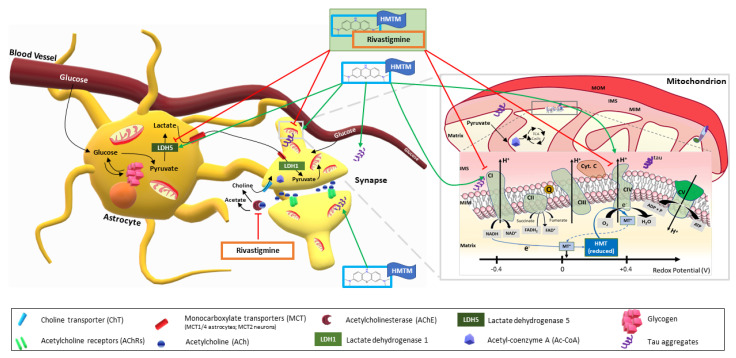
Proposed mechanism of action of HMTM in brain energy metabolism and interference by the acetylcholinesterase inhibitor rivastigmine. HMTM mono-dosing increases the levels of LDH-A subunits, the sole components of the astrocytic LDH5 isoenzyme responsible for converting pyruvate into l-lactate. l-Lactate is transported to the neurons via the monocarboxylate transporters, where it is converted back to pyruvate by the neuronal LDH1 (tetramer isoenzyme composed solely of LDH-B subunits). Pyruvate is the precursor for the production of acetyl-coenzyme A (Acetyl-CoA), which is the primary substrate initiating the TCA cycle in the mitochondrial matrix. The TCA cycle produces reducing equivalents in the forms of NADH and FADH_2_ that are subsequently oxidised by the mitochondrial ETC, initiating the electron flow that generates the transmembrane potential that drives the production of ATP. The MT moiety in its oxidised form (MT^+^) has a redox potential of 10 mV, which positions this chemical entity favourably to carry electrons directly from the reducing equivalents at the level of complex I to complex IV, therefore facilitating electron transport. MT will undergo continuous cycles of reduction and oxidation during the process of accepting and donating electrons. The pharmacological action of rivastigmine is to inhibit acetylcholinesterase, increasing the levels of acetylcholine, which clinically results in transient but limited improvement of AD symptoms. We show here that rivastigmine also prevents HMTM-induced increase in the levels of LDH-A, putatively affecting mostly astrocytic LDH5, resulting in a defective l-lactate supply to the neurons and ultimately decreasing production of ATP through oxidative phosphorylation, which mostly neurons are recognised to be mainly dependent on for highly energy-demanding process of neurotransmission. Red arrows imply inhibitory effects of the combination of HMTM and rivastigmine; green arrows represent enhancing effects of HMTM.

We propose that HMTM exerts a compartmentalised dual cell-specific effect, decreasing the levels of l-lactate despite a putative increase in the production of astrocytes through an increase in the levels of LDH-A (Figure 6). Given the predominance of neuronal mitochondrial oxidative phosphorylation over the glycolytic profile of astrocytes [65,66], the imbalance between astrocytic LDH-A and l-lactate levels, along with HMTM-enhanced mitochondrial activity, leads us to suggest that l-lactate is likely being transported to neurons through the astrocyte–neuron lactate shuttle. In neurons, l-lactate is used to produce acetyl-CoA, fuelling the TCA cycle and, ultimately, oxidative phosphorylation (Figure 6). Whereas the chronic administration of rivastigmine prior to HMTM dosing further reduced the levels of l-lactate, it failed to induce an increase in the levels of LDH-A (Figure 6). In balance, the failure to induce LDH-A indicates that the drug combination is less likely to stimulate the astrocyte–neuron lactate shuttle and is even less likely to result in an enhanced neuronal energy metabolism since, under this drug-dosing regimen, mitochondrial activity is not improved. Our group recently showed in a large-scale proteomics study performed on another mouse model of tauopathy—line 66 (L66), expressing the longest full-length human tau isoform carrying a P301S mutation—that out of 2000 spots from 2-DE analysed by MS, 3.5% were dysregulated in L66, with 22% of those belonging to metabolic pathways and regulated by HMTM [78]. From a pathway analysis, it was proposed that while metabolic dysfunction in general is likely corrected by HMTM due to action of HMTM as a TAI, the HMTM-mediated improvement of the mitochondrial oxidative phosphorylation may be independent of tau dissolution [78]. This agrees with the previously reported ability of the MT moiety to facilitate electron transport [30,31]. Consequently, the underlying mechanism of drug interference most likely derives from a combination of the direct action of HMTM-enhancing cellular bioenergetics and from the direct inhibition of the aggregation of tau proteins. Therefore, the reduced availability of MT in the brain in the presence of rivastigmine may be related to interference with either or both mechanisms. In conclusion, the nature of the interference of rivastigmine with HMTM, from the perspective of the brain energy metabolism, stems from a dual cell-specific multiple-compartmentalised HMTM-induced facilitation of astrocytic trophic support of neurons in a landscape where mitochondrial activity is improved that is otherwise compromised by the acetylcholinesterase inhibitor due to a decrease in the availability of MT.

## Figures and Tables

**Table 1:** Summary of the number of experimental conditions initially planned and total number of mice (N) tested for each group. Riva, rivastigmine; HMTM, hydromethylthionine mesylate.

Experimental Groups			
Genotype	Treatment (mg/kg)		N
	Weeks 1–5	Weeks 6–11	
NMRI	Vehicle		14
NMRI	Riva 0.5	+Vehicle	14
NMRI	Vehicle	+HMTM 15	14
NMRI	Riva 0.5	+HMTM 15	12
L1	Vehicle		16
L1	Riva 0.1	+Vehicle	12
L1	Riva 0.5	+Vehicle	12
L1	Vehicle	+HMTM 5	10
L1	Vehicle	+HMTM 15	7
L1	Riva 0.1	+HMTM 5	12
L1	Riva 0.5	+HMTM 5	8
L1	Riva 0.1	+HMTM 15	6
L1	Riva 0.5	+HMTM 15	6

**Table 2:** Summary of the l-lactate and mitochondrial metabolic characterisation of the L1 transgenic mice in comparison with their respective wild-type control mice, NMRI. Data are expressed as median values ± IQR (*n* = 14–16). Differences are considered statistically significant when *p* < 0.05. LDH-A, lactate dehydrogenase subunit A; LDH-B, lactate dehydrogenase subunit B; NDUFB8-CI, NADH:ubiquinone oxidoreductase subunit B8-complex I; SDHB-CII, succinate dehydrogenase complex iron-sulphur subunit B-complex II; UQCRC2-CIII, ubiquinol-cytochrome c reductase core protein 2-complex III; MTCOI-CIV, mitochondrial cytochrome c oxidase subunit 1-complex IV; ATP5A-CV, ATP synthase F1 subunit alpha-complex V.

	NMRI (Median, IQR, *n* = 14)	L1 (Median, IQR, *n* = 16)	*p*
**l-Lactate** (µmol/mg protein)	0.144 (0.108–0.160)	0.202 (0.157–0.250)	0.00149
**LDH-A** (10^−2^; a.u.)	0.343 (0.230–0.518)	0.559 (0.449–0.660)	0.0275
**LDH-B** (10^−2^; a.u.)	0.165 (0.126–0.246)	0.252 (0.214–0.274)	0.0925
**NDUFB8-CI** (10^−5^; a.u.)	3.01 (2.41–4.06)	3.33 (2.54–3.93)	0.919
**SDHB-CII** (10−5; a.u.)	2.26 (1.51–3.31)	2.64 (1.74–3.75)	0.473
**UQCRC2C-CIII** (10^−5^; a.u.)	4.62 (4.35–5.85)	4.82 (4.29–5.88)	0.790
**MTCOI-CIV** (10^−5^; a.u.)	3.26 (2.40–4.05)	3.28 (3.08–4.58)	0.448
**ATP5A-CV** (10^−5^; a.u.)	3.01 (2.38–3.28)	3.77 (2.96–4.36)	0.142
**Complex I activity** (normalised to citrate synthase)	0.457 (0.303–0.664)	0.408 (0.226–0.563)	0.401
**Complex IV activity** (normalised to citrate synthase)	1.41 (1.05–2.23)	1.62 (1.13–2.09)	0.886

## Data Availability

The data that support the findings of this study are available from the corresponding author, C.M.W., upon reasonable request.

## Supplementary Materials

The following supporting information can be downloaded at: https://www.mdpi.com/article/10.3390/biomedicines10040867/s1, Figure S1: Relative levels of mitochondrial complex I (**A**) and complex IV (**B**) activity in the brains of NMRI background controls and L1 mice under HMTM mono- and add-on dosing regimens to rivastigmine; Figure S2: Concentration of MT in the brains of NMRI control and L1 mice; Figure S3: Relative levels of subunits of the mitochondrial electron transport chain (ETC) respiratory complexes in the brains of NMRI controls and L1 mice; Figure S4: Levels of l-lactate in the brain tissue of NMRI controls and L1 mice; Figure S5: Relative levels of LDH-A (**A**) and LDH-B (**B**) in the brains of NMRI controls and L1 tau transgenic mice; Figure S6: Concentration of MT in the plasma of NMRI control and L1 mice.
