# Supplementary material for: HMTM-Mediated Enhancement of Brain Bioenergetics in a Mouse Tauopathy Model Is Blocked by Chronic Administration of Rivastigmine
Source: Biomedicines. 2022 Apr 7;10(4):867. doi: 10.3390/biomedicines10040867 (PMC9029156; doi:10.3390/biomedicines10040867)
Supplement: Supplementary file 1 [file biomedicines-10-00867-s001.zip › biomedicines-1668349-supplementary.pdf]

biomedicines

MDPI

Supplementary Material

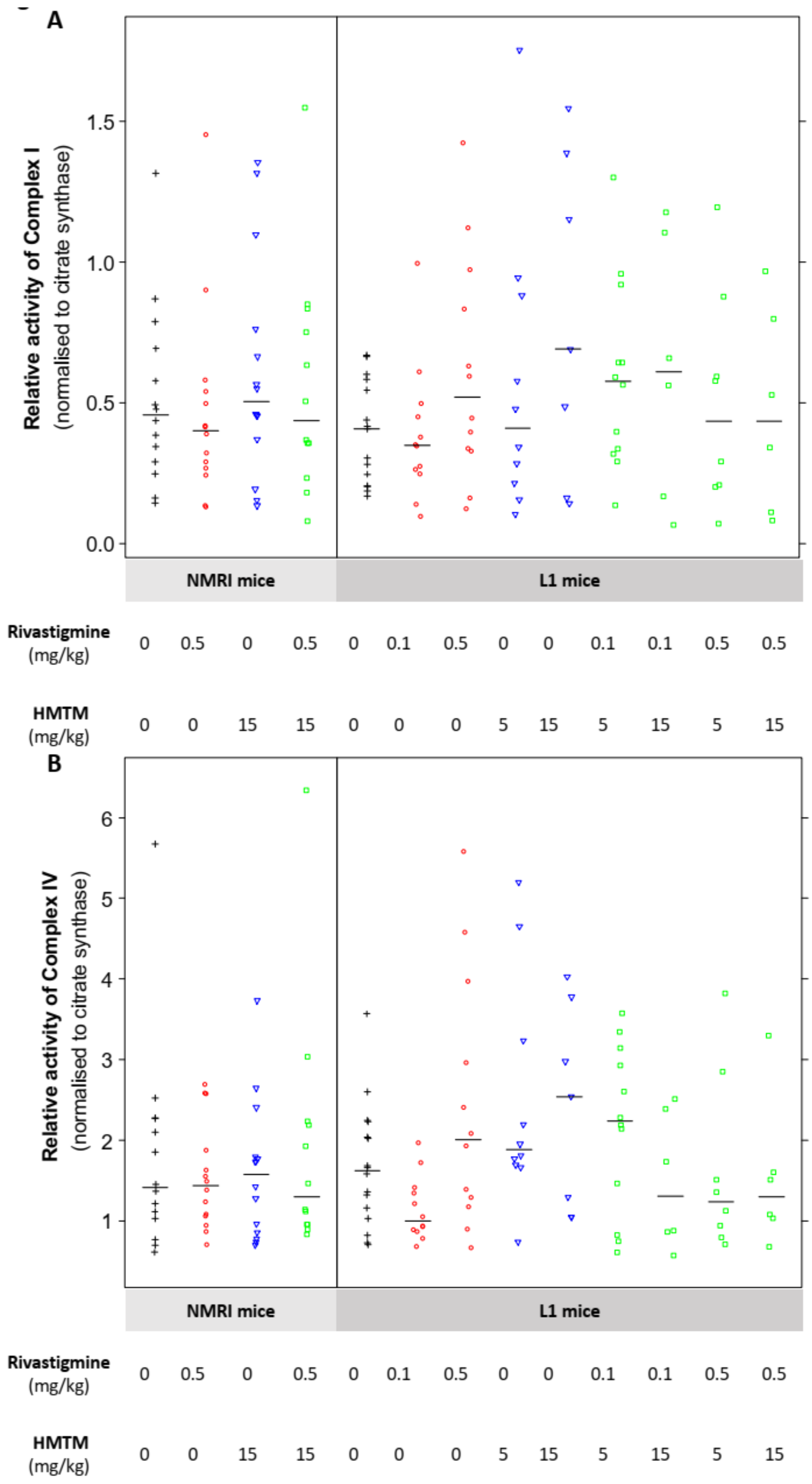

Figure S1. Relative levels of mitochondrial complex I (**A**) and complex IV (**B**) activity in the brains of NMRI background controls and L1 mice under HMTM mono- and add-on dosing regimens to rivastigmine. Activities are normalized to the activity of citrate synthase (CS) and data are expressed as median values ± IQR (n = 6–16). Regardless of the doses, the black symbols represent the vehicle group; the red symbols represent the rivastigmine-dosed mice; the blue symbols represent the experimental groups dosed with HMTM; and the green symbols represent the groups dosed with both HMTM and rivastigmine.

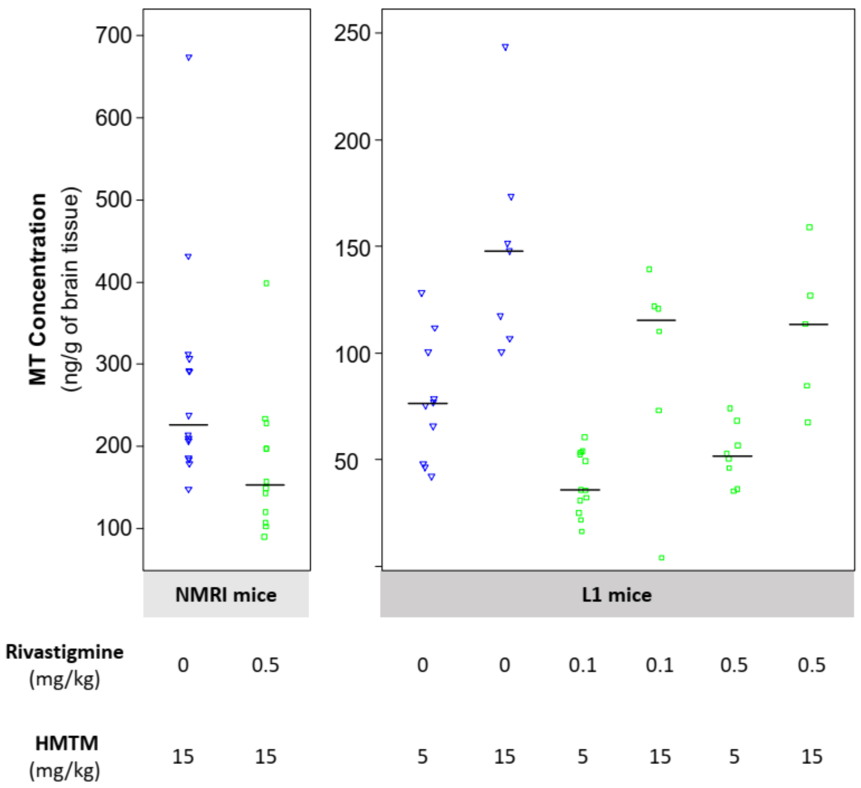

Figure S2. Concentration of MT in the brains of NMRI control and L1 mice. Data are expressed as median ± IQR (n = 5–14). Regardless of the doses, the blue symbols represent the experimental groups dosed with HMTM; and the green symbols represent the groups dosed with both HMTM and rivastigmine.

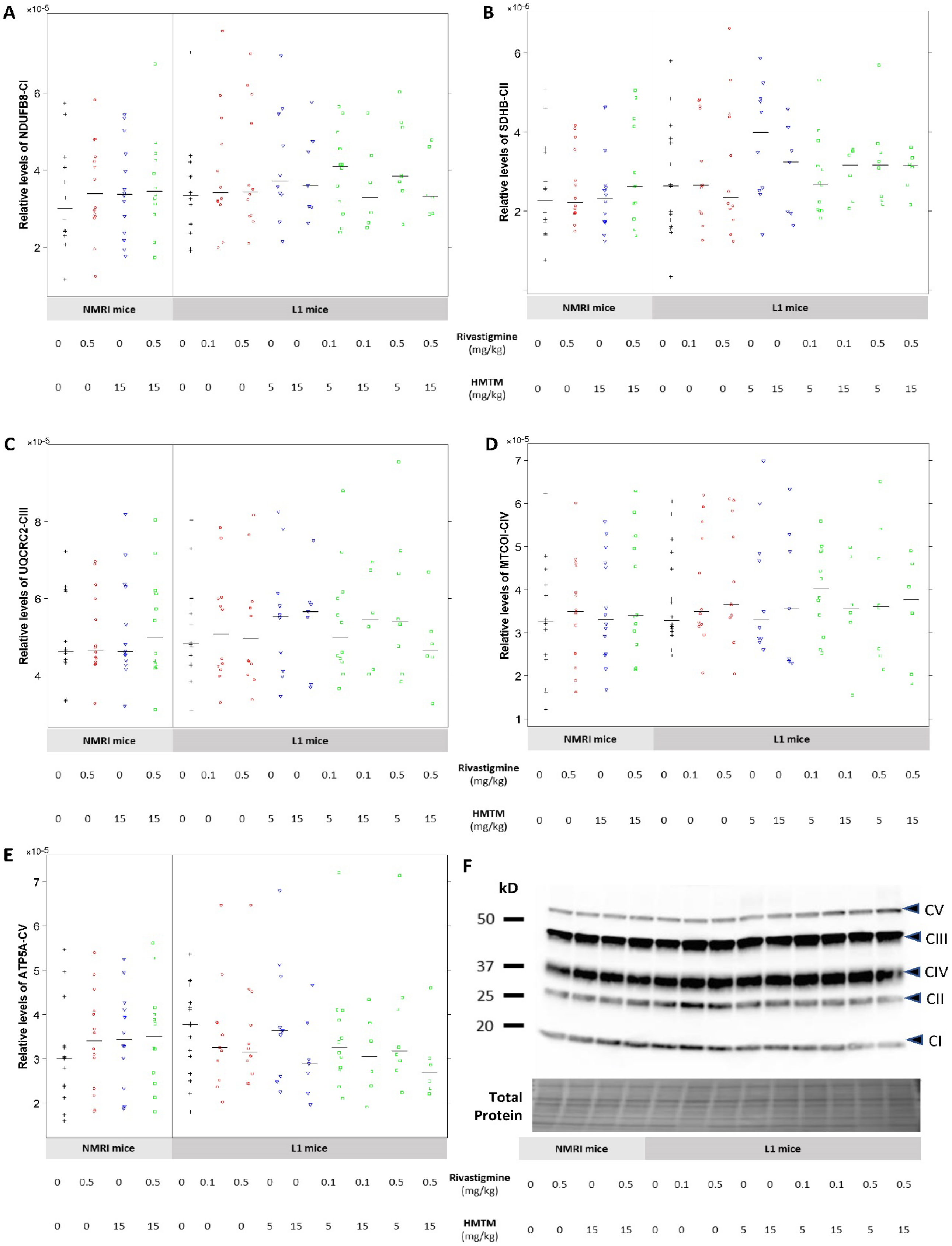

Figure S3. Relative levels of subunits of the mitochondrial electron transport chain (ETC) respiratory complexes in the brains of NMRI controls and L1 mice. None of the experimental conditions altered the levels of (**A**) complex I (CI) NADH:ubiquinone oxidoreductase subunit B8 (NDUFB8), (**B**) complex II (CII) succinate dehydrogenase complex iron-sulphur subunit B (SDHB), (**C**) complex

III (CIII) ubiquinol-cytochrome C reductase core protein 2 (UQCRC2), (**D**) complex IV (CIV) mitochondrial cytochrome c oxidase subunit 1 (MTCOI), or (**E**) complex V (CV) ATP synthase F1 subunit alpha (ATP5A). (**F**) Representative immunoblot for the detection of the ETC complexes in the total protein extracts from the brains of mice, and respective total protein labelling for samples. Data are expressed as median values ± IQR (*n* = 6–16). Regardless of the doses, the black symbols represent the vehicle group; the red symbols represent the rivastigmine-dosed mice; the blue symbols represent the experimental groups dosed with HMTM; and the green symbols represent the groups dosed with both HMTM and rivastigmine.

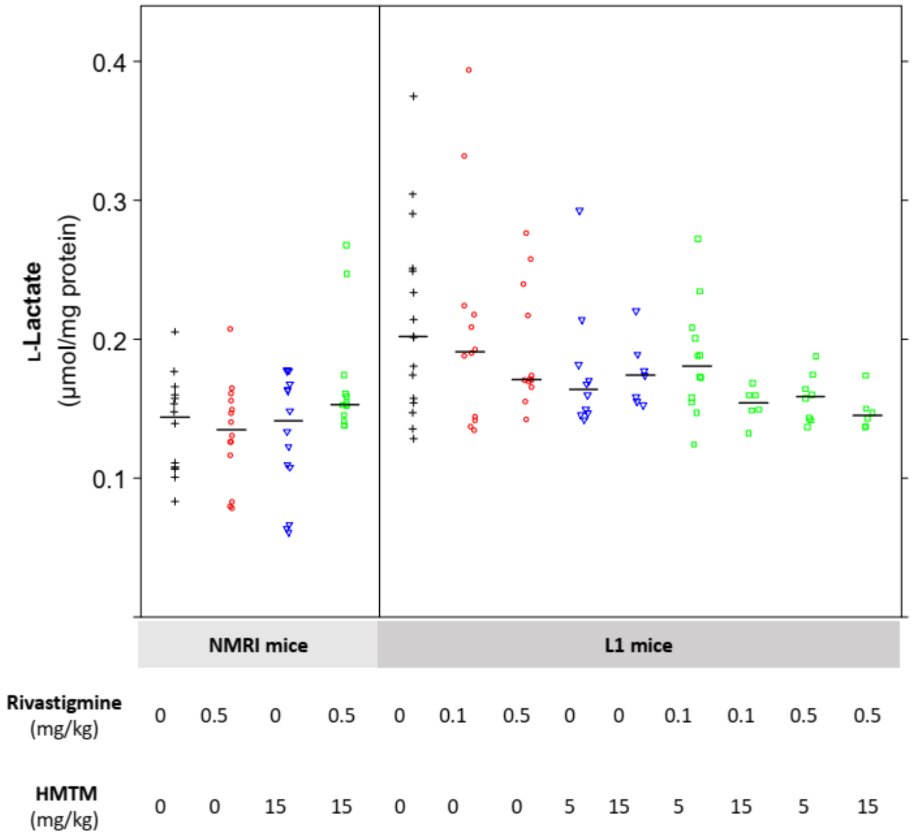

Figure S4. Levels of L-lactate in the brain tissue of NMRI controls and L1 mice. Data are expressed as median ± IQR (*n* = 6–16). Regardless of the doses, the black symbols represent the vehicle group; the red symbols represent the rivastigmine-dosed mice; the blue symbols represent the experimental groups dosed with HMTM; and the green symbols represent the groups dosed with both HMTM and rivastigmine.

(**A**)

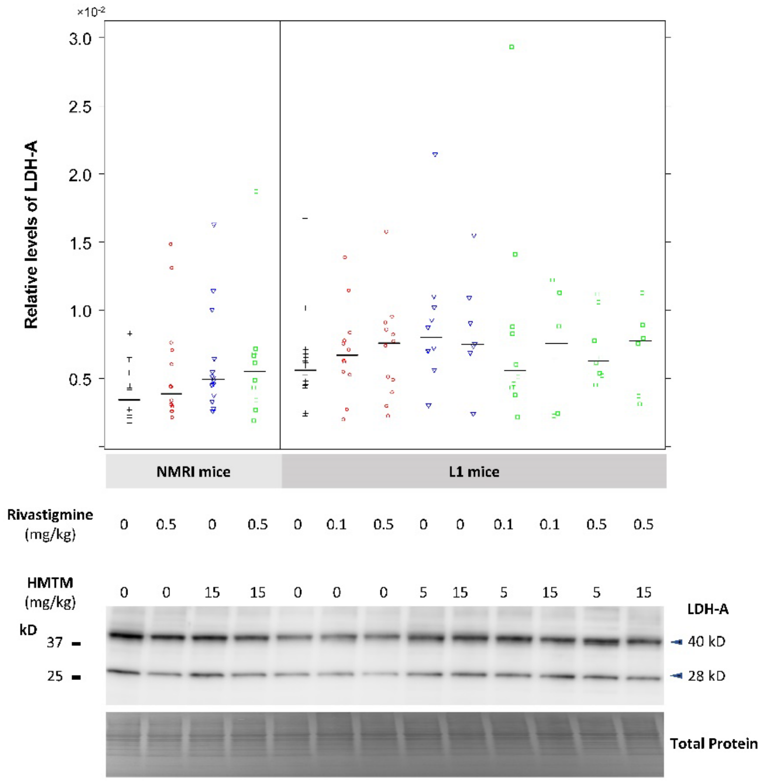

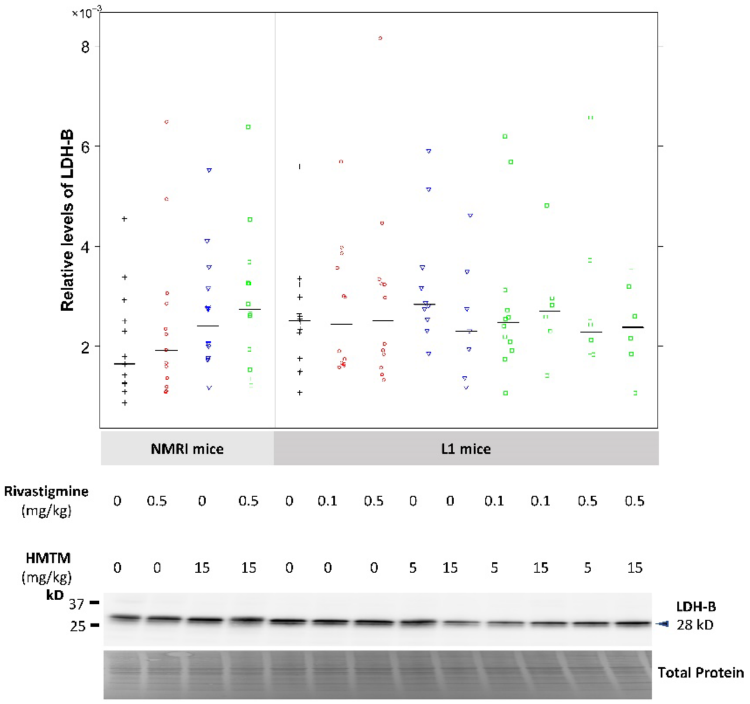

(**B**)

Figure S5. Relative levels of LDH-A (**A**) and LDH-B (**B**) in the brains of NMRI controls and L1 tau transgenic mice. Accompanied by image of representative immunoblot. Data are expressed as median values ± IQR (n = 6–16). Regardless of the doses, the black symbols represent the vehicle group; the red symbols represent the rivastigmine-dosed mice; the blue symbols represent the experimental groups dosed with HMTM; and the green symbols represent the groups dosed with both HMTM and rivastigmine.

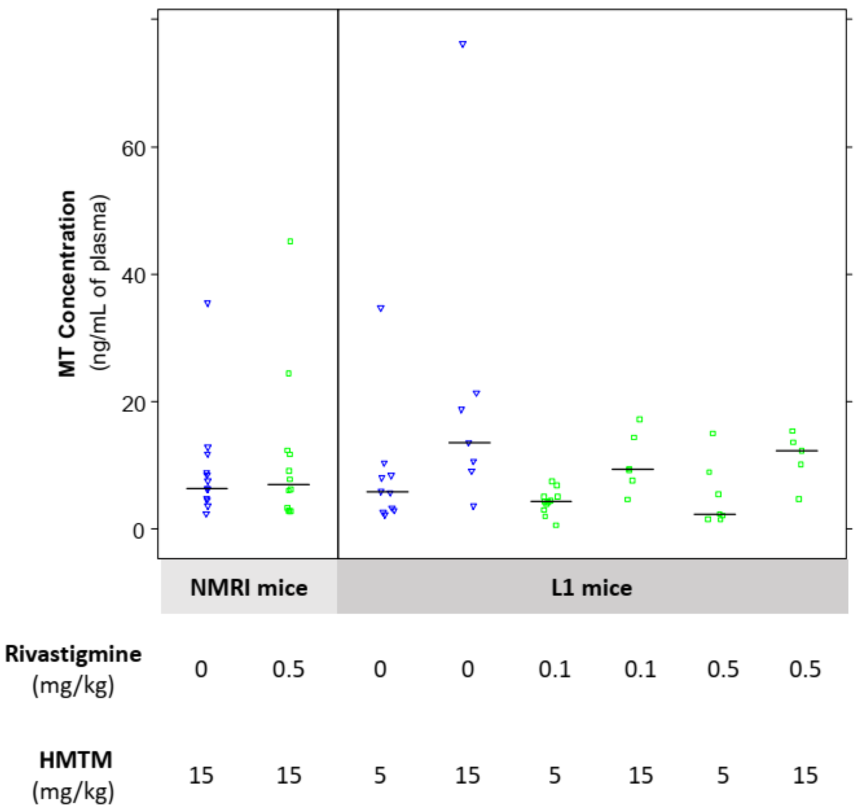

Figure S6. Concentration of MT in the plasma of NMRI control and L1 mice. Data are expressed as median ± IQR (n = 5–14). Regardless of the doses, the blue symbols represent the experimental groups dosed with HMTM; and the green symbols represent the groups dosed with both HMTM and rivastigmine.
